# Adverse Birth and Child Outcomes in Children Fathered by Men Treated with Antidiabetics Prior to Conception: A Nationwide Cohort Study

**DOI:** 10.3390/jcm11216595

**Published:** 2022-11-07

**Authors:** Bente Mertz Nørgård, Jens Fedder, Line Riis Jølving, Per Damkier, Jan Nielsen

**Affiliations:** 1 Center for Clinical Epidemiology, Odense University Hospital, 5000 Odense, Denmark; 2 Research Unit of Clinical Epidemiology, Department of Clinical Research, University of Southern Denmark, 5000 Odense, Denmark; 3 Centre of Andrology and Fertility Clinic, Odense University Hospital, 5000 Odense, Denmark; 4 Research Unit of Gynecology and Obstetrics, Department of Clinical Research, University of Southern Denmark, 5000 Odense, Denmark; 5 Department of Clinical Pharmacology, Odense University Hospital, 5000 Odense, Denmark; 6 Department of Clinical Research, University of Southern Denmark, 5000 Odense, Denmark

**Keywords:** birth outcomes, clinical epidemiology, diabetes mellitus, insulin, non-insulin anti-hyperglycemic agents, paternal, reproduction, type 1 diabetes, type 2 diabetes

## Abstract

Background: The safety of fathers’ use of antidiabetic drugs in terms of child outcomes is an important clinical question. We aimed to assess the risk of adverse birth and early childhood outcomes after fathers’ use of antidiabetics prior to conception. Methods: A nationwide cohort study based on Danish health registries. The study comprised all live born singleton children in Denmark (1997 through 2018). Children were categorized according to fathers’ filled prescriptions for antidiabetic drugs three months prior to conception. Exposed cohorts: children born after paternal use of insulin or non-insulin anti-hyperglycemic agents. The unexposed constituted children born by fathers not treated with antidiabetics prior to conception. We examined adverse birth outcomes (preterm birth, small for gestational age (SGA)), and adverse childhood outcomes in the first year of life (major congenital malformations (MCMs), and infections diagnosed at a hospital). Results: A total of 1,318,684 children were included. In all, 5527 children were born after paternal use of insulin, 2121 after use of non-insulin anti-hyperglycemic agents, and 1,311,036 were unexposed. After fathers’ use of insulin we did not find increased risk of adverse outcomes. After fathers’ use of metformin, the adjusted OR of MCMs was 1.40 (95% CI 1.11–1.76). After fathers’ use of sulfonylureas, the adjusted OR of SGA was 1.80 (95% CI 1.11–2.93), and for child gastrointestinal infections the adjusted HR was 1.76 (95% CI 1.04–2.99). Conclusions: Fathers’ use of insulin was reassuring. Metformin and sulfonylureas were associated with selected adverse outcomes. Our findings suggest an additional 14 MCMs per 1000 fathers exposed to metformin prior to conception. As there is no meaningful supporting biological rationale, these findings should be confirmed in a different population prior to clinical consequences being drawn.

## 1. Introduction

The association between maternal factors and the health of the newborn child has attracted significant attention during decades, but the paternal contributions have often not been studied. Fathers’ health and fathers’ use of medications prior to conception may have significant importance on the health of the offspring, and accumulating evidence has shown that paternal exposures may induce genetic and epigenetic alternation in sperm, which in turn increases the risk of adverse health outcomes in the offspring [1,2,3,4,5]. Paternal preconception factors such as stress, diet, obesity, and toxins have thus been associated with major congenital malformations (MCMs), cancers, impaired growth, obesity, and metabolic risk factors in the offspring [1,5,6,7,8,9]. Paternal preconception drug use may thus affect child outcomes. 

The association between paternal use of antidiabetics prior to conception and the risk of different adverse child outcomes has not been examined. Only one recent study has examined preconception paternal use of antidiabetics and MCMs, and suggested that metformin was associated with MCMs [10]. The relevance for studying the impact of paternal use of antidiabetics on the health of the offspring is significant as diabetes is one of the major problems threatening human health, and the diabetes incidence is increasing in men at the reproductive age [11,12,13]. Former studies on diabetic men have not focused on the health of the offspring, but on other reproductive aspects. It is thus well known that diabetes has significant negative impact on functional sperm characteristics [11,12,13,14,15], and that diabetes causes erectile dysfunction [16,17], impotence, retrograde ejaculation, and decreased sexual desire [17,18,19,20,21], as well as abnormalities in testicular functions and disruption of the spermatogenesis endocrine control, leading to an abnormal sperm production [12,13]. It is a central question, but so far unsettled, whether non-insulin anti-hyperglycemic agents may negatively influence the offspring. It is known that metformin may reduce testosterone levels independently of glycemic control [22], and a comprehensive review has concluded that adverse effects of metformin in the germ cell populations of offspring exposed in utero and those of subsequent generations are not clear [23].

We hypothesized that paternal use of antidiabetic drugs prior to conception might have negative consequences for offsprings’ health, i.e., adverse birth outcomes and diseases during early childhood. Regarding adverse birth outcomes, the most important outcomes that predict morbidity and mortality later in life, are preterm birth, impaired fetal growth, and MCMs. Regarding diseases during childhood, infections are the most common occurring diseases. Therefore, based on nationwide Danish data, we examined the associations between paternal use of insulin or non-insulin anti-hyperglycemic agents prior to conception and adverse birth outcomes (preterm birth and small for gestational age (SGA)), and adverse child outcomes in the first year of life (MCMs and infections). 

## 2. Materials and Methods

### 2.1. Design and Setting 

This observational cohort study is based on nationwide Danish health registries. The Danish population includes approximately 5.8 million people, >90% Caucasians, and all citizens have access to a tax supported health care system. The uniform organization of the health care system allowed us to use a population-based design where we used the power of combining information across nationwide health registries. We used data from (i) the Medical Birth Registry which has recorded information on all births in Denmark since 1973 and includes data on the mother, the father, pregnancy-related information, and information on birth outcomes [24,25], (ii) the Danish National Patient Registry which includes information on all hospital contacts for the entire Danish population since 1977 (including date of admission and discharge, procedures performed, and diagnoses based on the International Classification of Diseases (ICD-8 before 1994 and ICD-10 from 1994 onward) [26], (iii) the nationwide Prescription Registry which includes data on all filled prescriptions in Denmark since 1 January 1995. All pharmacies in Denmark are equipped with computerized accounting systems which send key data to the Prescription Registry, and all drugs are classified according to the anatomical therapeutical chemical (ATC) classification system [27], and (iv) the Civil Registration System which includes information on death and immigration [28]. The assignment of a unique civil registration number to each citizen at birth or immigration provides an exceptional system for valid record linkage on an individual level. 

### 2.2. Study Population

The population comprised all live born singleton children, born in Denmark from 1 January 1997, until 31 December 2018, identified in the Medical Birth Registry together with the registered father and mother of the child. 

### 2.3. Exposed Cohorts

For the children in the study population, we linked information on preconception paternal use of antidiabetic drugs based on filled prescriptions. We identified paternal use of insulin (ATC A10A), or non-insulin anti-hyperglycemic agents (ATC A10B), within a period of three months prior to the date of conception. The calculation of the date of conception was based on information from either estimated or ultrasound measured gestational age in the Medical Birth Registry. 

*Exposed cohort 1* included children fathered by men who received insulin at least once within three months prior to the date of conception. In this cohort the father was not allowed to have had concomitant or prior use of non-insulin anti-hyperglycemic agents.

*Exposed cohort 2* included children fathered by men who received non-insulin anti-hyperglycemic agents at least once within three months prior to the date of conception. In this cohort the father was not allowed to have had concomitant or prior use of insulin. For non-insulin anti-hyperglycemic agents we retrieved information on types (metformin A10BA, sulfonylureas A10BB, and other types of non-insulin anti-hyperglycemic agents).

### 2.4. Unexposed Cohort

From the study population, the unexposed cohort consisted of all children fathered by men who were not treated with insulin or non-insulin anti-hyperglycemic agents three months prior to the conception. 

### 2.5. Birth and Child Outcomes 

The birth outcomes included preterm birth (birth before 37 completed weeks of pregnancy), and birthweight as SGA, i.e., below the mean-2 SD according to gestational age and sex [29]. The child outcomes were MCMs and infections, all diagnosed within the first year of life. 

We had special exclusions when we examined MCMs. We excluded mothers who filled prescriptions (30 days before conception or during pregnancy) for drugs suspected to be teratogens (retinoids, angiotensin-converting enzyme inhibitors, vitamin K antagonists, valproic acid, lithium, carbamazepine, oxcarbazepine, phenytoin, phenobarbital or methotrexate) [30,31]. Fathers who filled prescriptions to methotrexate within three months prior to conception were also excluded due to an adverse effect of methotrexate on spermatogenesis, primarily affecting the germ cells [32,33,34]. MCMs were identified in the Danish National Patient Registry, ICD-10 codes from chapter Q (congenital malformations, deformations, and chromosomal abnormalities) and D18.1A (Cystic hygroma), D21.5 (Sacral teratoma), and D82.1 (Pharyngeal pouch syndrome) in line with EUROCAT criteria. MCMs were identified by excluding minor malformations based on the EUROCAT’s classification of malformations [35,36]. Child infections were identified in the Danish National Patient Registry, i.e., all infections were diagnosed in a hospital setting. Mild infections diagnosed by a general practitioner were thus not included in the outcome assessment. The types of infections were given according to organ systems (ICD-10 groups): respiratory tract, gastrointestinal tract, urological/gynecological, skin and subcutaneous tissue, bacteremia, and other infections. 

### 2.6. Confounders 

The Medical Birth Registry gave information on paternal and maternal age (≤30 years, 31–40 years, ≥41 years) at the time of child birth, sex of the child, parity (1/>1 pregnancy), maternal body mass index (BMI) at the first antenatal visit, maternal smoking (yes/no), calendar year of birth (1997–2002, 2003–2008, 2009–2014, 2015–2018), maternal and paternal Charlson’s Comorbidity Index (excluding diabetes from the index) [37], and maternal use of antidiabetics at any given time prior to or during pregnancy.

### 2.7. Statistical Analyses

A contingency table is given for the main study variables according to the exposed and unexposed cohorts. A significance level of 0.05 was chosen; estimates outside the 95% confidence interval are regarded as statistically significant. We estimated the risk of preterm birth and SGA in logistic regression analyses, with robust variance estimation, accounting for multiple children by the same parents. We computed crude and adjusted prevalence odds ratios (OR) with 95% confidence intervals (CI) following paternal use of insulin prior to conception relative to the unexposed cohort. Similarly, we computed the crude and adjusted ORs for preterm birth and SGA following paternal use of non-insulin anti-hyperglycemic agents prior to conception (stratified according to metformin, sulfonylureas, and other types of non-insulin anti-hyperglycemic agents). When we examined the time to event outcomes, i.e., MCMs and the risk of infections within the first year of life, the follow up of the children started on the date of live birth and ended on the date of the first diagnosis of the specific outcome of interest, emigration, death, or the first birthday of the child, whichever came first. To analyze whether the offspring MCMs or infections were different between the exposed and unexposed cohort, we used a Cox proportional hazard regression model estimating the hazard ratios (HRs) for each outcome, with robust variance estimation accounting for clustering of multiple children born by the same parents. Types of infections were estimated and also the overall risk of infection by estimating the risk of a first infection (regardless of type). We reported the HR with 95% CI and inspected the Cox proportional hazard assumption graphically. 

We used two regression models adjusting for confounders. In model A we adjusted for maternal and paternal age, maternal and paternal comorbidity, mother’s use of antidiabetics, parity, sex of the child, and calendar year. Additionally, we added maternal BMI and maternal smoking in model B. When we examined the impact of a specific type of non-insulin anti-hyperglycemic agents, the models also included the use of other types of non-insulin anti-hyperglycemic agents prior to conception. 

For outcome categories with less than 5 child outcomes in the exposed cohort we did not calculate crude or adjusted HR’s due to lack of statistical precision. 

### 2.8. Approvals and Ethics 

The study was approved by the Danish Data Protection Agency (j.nr. 20/4674). According to Danish law, no ethical approvals of register-based studies are required. 

### 2.9. Patient and Public Involvement

Patient representatives are part of the research council at our department, and they have been involved in parts of the research process. They have contributed to the discussion about the study idea and which outcomes to measure. The patient representatives were not involved in the design of the study, analyses, or in the writing of the paper. 

## 3. Results

Table 1 shows the characteristics of the study cohorts. A total of 1,318,684 children were included. Of these, 5527 children were fathered by men who used insulin prior to conception, and 2121 children were fathered by men who filled prescription for non-insulin anti-hyperglycemic agents prior to conception. The most commonly used type of non-insulin anti-hyperglycemic agent was metformin (76.2%). A total of 1,311,036 children were fathered by unexposed men. Fathers treated with non-insulin anti-hyperglycemic agents were older and had more comorbid diseases than fathers treated with insulin (54.6% vs. 12.0% in age group +41 years, and 17.2% vs. 8.8% in comorbidity). Fathers not treated with either insulin or non-insulin anti-hyperglycemic agents were similar to fathers treated with insulin as regards age and comorbidity.

### 3.1. Preconception Paternal Use of Insulin

Compared to the unexposed, the adjusted OR of preterm birth and SGA in children fathered by men treated with insulin prior to conception was 1.10 (95% CI 0.95–1.27) and 0.96 (95% CI: 0.78–1.18), respectively (Table 2). The adjusted HR of MCMs was 0.97 (95% CI: 0.82–1.15), and the overall adjusted risk of infections was 0.99 (95% CI 0.91–1.08) (Table 2). There were no statistically significantly increased risks of subtypes of child infections (Table 2).

### 3.2. Preconception Paternal Use of Non-Insulin Anti-Hyperglycemic Agents

**Figure 1:**
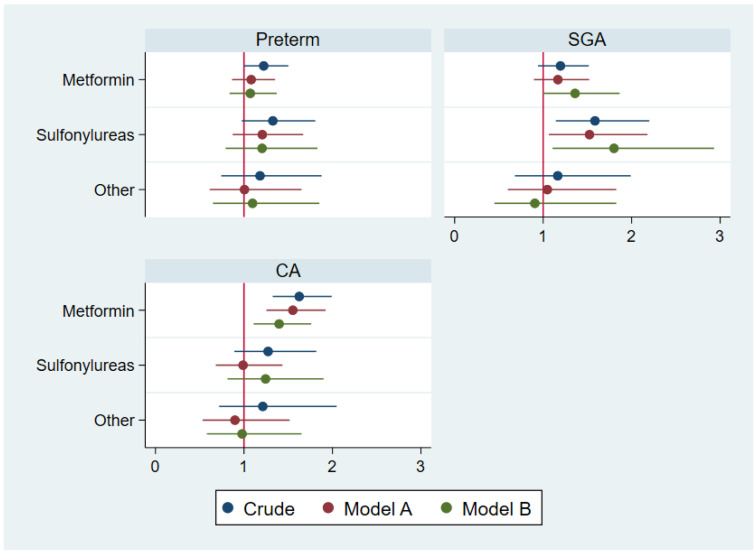
Shows the crude and adjusted ORs for preterm birth and small for gestational age (SGA), and the HRs for congenital abnormalities, according to paternal use of non-insulin anti-hyperglycemic agents (metformin, sulfonylureas, others) prior to conception. In model A, we adjusted for maternal and paternal age, maternal and paternal comorbidity, mother’s use of antidiabetics, parity, sex of the child, calendar year, and other types of non-insulin anti-hyperglycemic agents prior to conception. In model B, we added maternal BMI and maternal smoking.

The crude and adjusted ORs/HRs for preterm birth, SGA, and MCMs according to paternal use of specific types of non-insulin anti-hyperglycemic agents prior to conception are given in Figure 1. For metformin, there was no increased risk of preterm birth, and the adjusted OR of SGA was 1.36 (95% CI 0.99–1.86). The crude HR of MCMs after paternal use of metformin was 1.62 (95% CI 1.32–1.99), and 1.40 (95% CI 1.11–1.76) in the full regression model (Table 3). This corresponds to an additional 14 MCMs per 1000 fathers exposed to metformin prior to conception. We found that the types of MCMs were distributed across different organ systems (the four most common categories were heart defects 29 (32%), genital 20 (22%), limb 15 (16%), digestive 10 (11%)). In the children fathered by men using metformin, the details according to maternal age were: median age 33 years and 25–75% percentile 30–37 years. 

**Figure 2:**
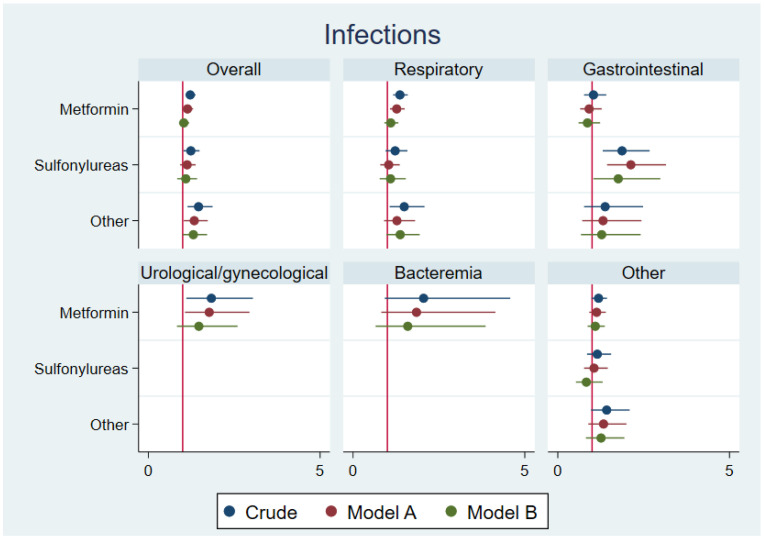
Shows the HRs for overall child infections, and types of infections in the first year of life after paternal use of non-insulin anti-hyperglycemic agents (stratified according to metformin, sulfonylureas, others). In model A, we adjusted for maternal and paternal age, maternal and paternal comorbidity, mother’s use of antidiabetics, parity, sex of the child, calendar year, and other types of non-insulin anti-hyperglycemic agents prior to conception. In model B, we added maternal BMI and maternal smoking.

Figure 2 shows the HRs for child infections in the first year of life after paternal use of non-insulin anti-hyperglycemic agents. Metformin was not associated with an overall risk of infections, and when we examined specific types of infections, none were statistically significantly increased (Table 3). After paternal use of sulfonylureas, the adjusted ORs of preterm birth and SGA were 1.20 (95% CI 0.79–1.83) and 1.80 (95% CI 1.11–2.93), respectively (Table 3). The adjusted HR of MCMs was 1.24 (95% CI 0.81–1.90) (Table 3). The use of sulfonylureas was not associated with overall child infections, but with child gastrointestinal infections, adjusted HR 1.76 (95% CI 1.04–2.99), Table 3.

## 4. Discussion

In this population-wide registry-based study, children fathered by men treated with insulin prior to conception did not have an increased risk of preterm birth or SGA, and within the first year of life no increased risk of MCMs or infections was diagnosed at hospital. We found that paternal use of metformin was associated with a 1.4-fold increased risk of MCMs, but we did not have the power to examine subtypes of MCMs. Paternal use of sulfonylureas was associated with a 1.80-fold increased risk of SGA, and a 1.76-fold increased risk of gastrointestinal child infections. 

No other study has examined the association between paternal use of antidiabetics and preterm birth, SGA, or childhood infections in the first year of life. Across all outcomes, our overall results were reassuring after paternal use of insulin and non-insulin anti-hyperglycemic agents, with the exception of the few associations related to metformin and sulfonylureas mentioned above. We do not know the underlying mechanisms for these findings, or whether the findings are causal or susceptible to confounding. Recently, an association between paternal use of metformin and MCMs has been suggested in another study from Denmark, reporting an adjusted OR of 1.40 (95% CI 1.08–1.82) [10]. Contrary to our analyses, that risk estimate was not adjusted for factors such as maternal BMI, maternal and paternal comorbidity, parity, and concomitant paternal use of other non-insulin anti-hyperglycemic agents. Nevertheless, we found a very similar adjusted OR (1.40, 95% CI 1.11–1.76). Our dataset has substantial overlapping data with the study by Wensink et al. [10], and therefore it is important to emphasize that our data cannot be used to validate an association between paternal use of metformin and MCMs. There are no biologically plausible explanations to support this finding. Based on animal models, the impact of metformin has been examined on factors such as testosterone level, testes antioxidant status, testes size, spermatogenesis index, sperm motility and concentration [38,39,40], and a comprehensive review concludes that an impact on the male reproductive system remains controversial [13]. One may speculate on effects potentially modulated through epigenetic changes in sperm cells, but there are no data on this [41]. A 40% increased risk corresponds to about 14 additional MCMs per 1.000 fathers receiving metformin prior to conception. While perhaps small in absolute numbers, this is clinically relevant with potentially substantial consequences to men of reproductive age receiving metformin. We therefore stress that our findings be reproduced in different populations before clinical consequences be made.

### Strengths and Limitations

The main strength of this study is the nationwide design which includes information on all singleton children during the study period. The data from the Danish heath registries are well-known tools for clinical epidemiological studies used in multiple studies, and are known for high data quality and high coverage [25,26,27]. We had access to the complete nationwide Prescription Registry, ensuring that all fathers were classified according to the drug exposure prior to conception, and the data are of high quality as a result of direct computerized transfer of information when a prescribed drug is dispensed at a pharmacy [27]. It is a strength that information on drug exposure was based on prescriptions and not on patient recall, which prevents recall bias. We obtained information on the child outcomes independently of the exposure and the hypothesis examined, thus preventing differential misclassification of the outcome. It is an important strength that we were able to adjust for several confounders. 

This study also has limitations. In an observational study like this, there is always a risk of unmeasured residual confounding, and we cannot rule out that some of our risk estimates are subject to uncontrolled confounding. We did not have data on socioeconomics or paternal BMI as no Danish health registry collects BMI on all men. Moreover, in this study based on health registries (N = 1,318,684 children), information on semen quality in all men is not feasible, and we did not have blood sugar levels of the fathers. In our analyses of MCMs, we excluded mothers who filled prescriptions for drugs suspected to be teratogens, but we cannot rule out an impact of other medications taken by the women during pregnancy. Secondly, it is difficult, or impossible, to isolate the effects of a drug from an effect of the underlying disease. However, the inability to distinguish an effect of diabetes from an effect of antidiabetic drugs is not a major problem in case of negative results, as associations indicating no increased risk can be interpreted to imply that the drugs have no harmful effects. Theoretically, if an impact of—for instance—insulin should have been examined, the reference population should include children born to fathers with type 1 diabetes not treated with insulin, and such a study will never be performed. Due to ethics, randomized trials are not designed to examine drug safety during pregnancy and conception, and therefore, clinical decisions on drug use during stages of gestation are based on evidence from observational studies, that might be vulnerable to bias and confounding. In case of results implying an increased risk of adverse outcomes it is relevant to discuss whether it is due to the drug or the underlying disease. We found that paternal use of some of the non-insulin anti-hyperglycemic agents was related to certain adverse outcomes, and we cannot rule out that these associations are caused by diabetes itself, or uncontrolled disease, rather than the drug. On the other hand, it cannot be ruled out that it is related to the drugs. Thirdly, in general there is a low prevalence of adverse birth outcomes, and therefore, sufficient statistical precision is always a matter of concern. This is particularly relevant for MCMs, because no known teratogens increase the risk of all MCMs, but rather tend to increase rates of selected MCMs. Ideally, the risk of specific MCMs should be examined, but this would have major implications on the sample size requirements. Therefore, we do not have the power to examine the risk of specific MCMs, and as cohort studies can only detect considerable increases in the risk of specific defects, most cohort studies are limited in their ability to provide assurance of safety.

## 5. Conclusions

The results of this cohort study suggest reassuring results for expecting fathers with diabetes, and the results are important for health professionals and for clinical counselling. Paternal treatment with insulin was not associated with adverse birth or important child outcomes in the first year of life. We found that paternal use of metformin or sulfonylureas was associated with an increased risk of a few selected adverse outcomes; these associations need to be confirmed in other datasets before clinical consequences are considered.

## Figures and Tables

**Table 1:** Descriptive characteristics of the study population according to exposure status. Fathers exposed to insulin (ATC: A10A), fathers exposed to non-insulin anti-hyperglycemic agents (ATC: A10B), and non-exposed fathers (study period from 1997 to 31 December 2018).

	Children Fathered by Men Treated with Insulin (ATC A10A) N = 5527	Children Fathered by Men Treated with Non-Insulin Anti-Hyperglycemic Agents (ATC A10B) N = 2121	Children Fathered by Men Not Treated with Insulin or Antidiabetics N = 1,311,036
Paternal age at child birth, years (n, %)			
−30	1585 (28.7)	103 (4.9)	400,628 (30.6)
31–40	3277 (59.3)	859 (40.5)	762,768 (58.2)
41–	665 (12.0)	1159 (54.6)	147,640 (11.3)
Paternal Charlson’s comorbidity index (n, %)			
0	5042 (91.2)	1756 (82.8)	1,230,252 (93.8)
>=1	485 (8.8)	365 (17.2)	80,784 (6.2)
Maternal use of antidiabetics (n, %)			
No	5437 (98.4)	2029 (95.7)	1,296,172 (98.9)
Yes	90 (1.6)	92 (4.3)	14,864 (1.1)
Maternal age at child birth, years (n, %)			
−30	2604 (47.1)	512 (24.1)	612,733 (46.7)
31–40	2760 (49.9)	1328 (62.6)	662,754 (50.6)
41–	163 (2.9)	281 (13.2)	35,549 (2.7)
Maternal Charlson’s comorbidity (n, %)			
0	5130 (92.8)	1963 (92.6)	1,227,353 (93.6)
>=1	397 (7.2)	158 (7.4)	83,683 (6.4)
Maternal smoking (n, %)			
No	4288 (77.6)	1778 (83.8)	1,002,296 (76.5)
Yes	831 (15.0)	239 (11.3)	180,175 (13.7)
Missing	408 (7.4)	104 (4.9)	128,565 (9.8)
Maternal BMI ^a^ (n, %)			
<18.5	155 (2.8)	48 (2.3)	37,420 (2.9)
18.5–24.9	2144 (38.8)	621 (29.3)	513,929 (39.2)
>=25	1367 (24.7)	1028 (48.5)	276,856 (21.1)
Missing	1861 (33.7)	424 (20.0)	482,831 (36.8)
Parity (n, %)			
First child	2433 (44.0)	582 (27.4)	570,606 (43.5)
Second or more	3028 (54.8)	1524 (71.9)	705,333 (53.8)
Missing	66 (1.2)	15 (0.7)	35,097 (2.7)
Child sex (n, %)			
Girl	2682 (48.5)	1032 (48.7)	638,060 (48.7)
Boy	2845 (51.5)	1089 (51.3)	672,976 (51.3)
Calendar year of birth (n, %)			
1997–2002	1512 (27.4)	300 (14.1)	377,636 (28.8)
2003–2008	1594 (28.8)	507 (23.9)	367,847 (28.1)
2009–2014	1469 (26.6)	726 (34.2)	336,183 (25.6)
2015–2018	952 (17.2)	588 (27.7)	229,370 (17.5)
Type of non-insulin anti-hyperglycemic agents ^b^ (n, %)			
Metformin		1617 (76.2)	
Sulfonylureas		664 (31.3)	
Others		327 (15.4)	
Father’s use of methotrexate prior to conception (n, %)	2 (0.0)	1 (0.0)	241 (0.0)
Mother’s exposure to known teratogens ^c^ (n, %)	39 (0.7)	14 (0.7)	4053 (0.3)

(a) BMI = body mass index. (b) Adds up to more than 100% as some children were born to fathers who filled prescription for more than one type of non-insulin anti-hyperglycemic agent prior to conception. Type of non-insulin anti-hyperglycemic agents are only relevant information for the cohort of children fathered by men using non-insulin anti-hyperglycemic agents prior to conception. (c) Mother’s use of the following drugs within three months before conception or during pregnancy: retinoider, angiotensein-converting enzyme inhibitors, vitamin K antagonists, valproic acid, lithium, carbamazepine, oxcarbazepine, phenytoin, phenobarbital, and methotrexate.

**Table 2:** Crude and adjusted odds ratio (OR), with 95% confidence interval (CI), for preterm birth and small for gestational age (SGA) in live born singletons fathered by men treated with insulin before conception, and the crude and adjusted hazard ratios (HR) for congenital abnormalities and early life infections in live born singletons fathered by men treated with insulin before conception.

	Children Fathered by Men Treated with Insulin within Three Months before Conception		Children Fathered by Men Not Treated with Insulin or Non-Insulin Anti-Hyperglycemic Agents within Three Months before Conception				
	Events (%)	N	Events (%)	N	Crude OR (95% CI)	Adjusted OR ^a^ (95% CI)	Adjusted OR ^b^ (95% CI)
**BIRTH OUTCOMES**							
Preterm birth	299 (5.4)	5527	65,050 (5.0)	1,311,036	1.10 (0.97–1.23)	1.06 (0.94–1.19)	1.10 (0.95–1.27)
SGA	164 (3.0)	5512	47,770 (3.7)	1,287,707	0.80 (0.68–0.93)	0.83 (0.71–0.97)	0.96 (0.78–1.18)
	**Events/total time at risk** **(years)**	**N**	**Events/total time at risk** **(years)**	**N**	**Crude HR** **(95% CI)**	**Adjusted HR ^a^** **(95% CI)**	**Adjusted HR ^b^** **(95% CI)**
**EARLY CHILD OUTCOMES (FIRST YEAR OF LIFE)**							
Major congenital malformations	189/5316	5486	46,692/ 1,261,546	1,306,743	0.96 (0.83–1.11)	0.94 (0.81–1.09)	0.97 (0.82–1.15)
Infections							
Overall infections	767/5132	5527	179,618/ 1,214,168	1,311,036	1.01 (0.94–1.08)	1.00 (0.93–1.07)	0.99 (0.91–1.08)
Respiratory	443/5301	5527	96,765/ 1,256,729	1,311,036	1.09 (0.99–1.19)	1.07 (0.97–1.17)	1.05 (0.94–1.17)
Gastrointestinal	125/5462	5527	32,017/ 1,290,404	1,311,036	0.92 (0.77–1.10)	0.91 (0.76–1.09)	0.94 (0.77–1.15)
Urological/gynecological	29/5497	5527	6644/ 1,300,267	1,311,036	1.03 (0.72–1.49)	1.03 (0.72–1.48)	1.02 (0.67–1.55)
Skin/subcutaneous tissue	0/5514	5527	121/ 1,304,014	1,311,036			
Bacteremia	11/5504	5527	2372/ 1,302,387	1,311,036	1.10 (0.61–1.99)	1.09 (0.60–1.98)	1.32 (0.71–2.46)
Other infections	305/5370	5527	74,234/ 1,268,781	1,311,036	0.97 (0.87–1.09)	0.96 (0.86–1.08)	0.93 (0.81–1.06)

(a) Adjusted for mother’s age (≤30 years, 31–40 years, ≥41 years), father’s age (≤30 years, 31–40 years, ≥41 years), parity (1 or more than 1), sex of the child, maternal and paternal comorbidity, calendar year of birth (1997–2002, 2003–2008, 2009–2014, 2015–2018), and mother’s use of antidiabetics in a regression model. (b) Adjusted for mother’s age (≤30 years, 31–40 years, ≥41 years), father’s age (≤30 years, 31–40 years, ≥41 years), parity (1 or more than 1), sex of the child, mother’s BMI (<18.5, 18.5–24.9, 25–29.9, ≥30, kg/m^2^) maternal smoking in pregnancy (yes/no), maternal and paternal comorbidity and calendar year of birth (1997–2002, 2003–2008, 2009–2014, 2015–2018) and mother’s use of antidiabetics in a regression model.

**Table 3:** Crude and adjusted odds ratio (OR), with 95% confidence interval (CI), for preterm birth and small for gestational age (SGA) in live born singletons fathered by men treated with non-insulin anti-hyperglycemic agents before conception, and the crude and adjusted hazard ratios (HR) for congenital abnormalities and early life infections in live born singletons fathered by men treated with non-insulin anti-hyperglycemic agents before conception. All analyses are stratified according to types of non-insulin anti-hyperglycemic agents (metformin, sulfonylureas, others).

	Children Fathered by Men Treated with Non-Insulin Anti-Hyperglycemic Agents within Three Months before Conception		Children Fathered by Men Not Treated with Insulin or Non-Insulin Anti-Hyperglycemic Agents within Three Months before Conception				
	Events (%)	N	Events (%)	N	Crude OR (95% CI)	Adjusted OR ^a^ (95% CI)	Adjusted OR ^b^ (95% CI)
**BIRTH OUTCOMES**							
**Preterm birth**							
Metformin	97 (6.0)	1617	65,080 (5.0)	1,311,540	1.22 (1.00–1.50)	1.08 (0.86–1.35)	1.07 (0.84–1.37)
Sulfonylureas	43 (6.5)	664	65,134 (5.0)	1,312,493	1.33 (0.97–1.81)	1.21 (0.87–1.67)	1.20 (0.79–1.83)
Other	19 (5.8)	327	65,158 (5.0)	1,312,830	1.18 (0.74–1.88)	1.01 (0.61–1.65)	1.10 (0.65–1.85)
**SGA**							
Metformin	71 (4.4)	1613	47,794 (3.7)	1,288,208	1.20 (0.94–1.52)	1.17 (0.90–1.52)	1.36 (0.99–1.86)
Sulfonylureas	38 (5.8)	660	47,827 (3.7)	1,289,161	1.59 (1.14–2.20)	1.52 (1.06–2.18)	1.80 (1.11–2.93)
Other	14 (4.3)	326	47,851 (3.7)	1,289,495	1.16 (0.68–1.99)	1.05 (0.60–1.83)	0.91 (0.45–1.83)
	**Events/total time at risk** **(years)**	**N**	**Events/total time at risk** **(years)**	**N**	**Crude HR** **(95% CI)**	**Adjusted HR ^a^** **(95% CI)**	**Adjusted HR ^b^** **(95% CI)**
**EARLY CHILD OUTCOMES (FIRST YEAR OF LIFE)**							
**Major congenital malformations**							
Metformin	92/1517	1603	46,704/1,262,036	1,307,246	1.62 (1.32–1.99)	1.55 (1.25–1.92)	1.40 (1.11–1.76)
Sulfonylureas	30/632	663	46,766/1,262,921	1,308,186	1.27 (0.89–1.82)	0.99 (0.68–1.43)	1.24 (0.81–1.90)
Other	14/310	325	46,782/1,263,243	1,308,524	1.21 (0.72–2.05)	0.90 (0.53–1.52)	0.98 (0.58–1.65)
**Infections**							
*Overall infections*							
Metformin	264/1465	1617	179,703/1,214,628	1,311,540	1.22 (1.08–1.38)	1.14 (1.00–1.30)	1.03 (0.89–1.19)
Sulfonylureas	110/602	664	179,857/1,215,491	1,312,493	1.24 (1.02–1.49)	1.13 (0.92–1.38)	1.09 (0.84–1.42)
Other	63/291	327	179,904/1,215,801	1,312,830	1.46 (1.14–1.87)	1.34 (1.03–1.73)	1.31 (1.00–1.71)
*Respiratory*							
Metformin	160/1525	1617	96,805/1,257,209	1,311,540	1.36 (1.17–1.59)	1.27 (1.07–1.50)	1.10 (0.91–1.32)
Sulfonylureas	59/626	664	96,906/1,258,109	1,312,493	1.22 (0.95–1.58)	1.04 (0.79–1.36)	1.09 (0.78–1.54)
Other	35/305	327	96,930/1,258,429	1,312,830	1.49 (1.07–2.08)	1.28 (0.90–1.81)	1.37 (0.97–1.94)
*Gastrointestinal*							
Metformin	41/1590	1617	32,040/1,290,897	1,311,540	1.04 (0.76–1.41)	0.91 (0.65–1.28)	0.86 (0.60–1.23)
Sulfonylureas	30/647	664	32,051/1,291,839	1,312,493	1.87 (1.31–2.67)	2.13 (1.44–3.15)	1.76 (1.04–2.99)
Other	11/322	327	32,070/1,292,165	1,312,830	1.38 (0.76–2.48)	1.32 (0.71–2.44)	1.28 (0.68–2.42)
*Urological/gynecological*							
Metformin	15/1601	1617	6644/1,300,768	1,311,540	1.83 (1.11–3.04)	1.77 (1.06–2.95)	1.47 (0.83–2.60)
Sulfonylureas ^c^	2/657	664	6657/1,301,712	1,312,493			
Other ^c^	1/325	327	6658/1,302,044	1,312,830			
*Skin/subcutaneous tissue*							
Metformin ^c^	1/1609	1617	121/1,304,515	1,311,540			
Sulfonylureas ^c^	0/659	664	122/1,305,466	1,312,493			
Other ^c^	0/325	327	122/1,305,799	1,312,830			
*Bacteremia*							
Metformin	6/1604	1617	2374/1,302,888	1,311,540	2.05 (0.92–4.58)	1.85 (0.82–4.15)	1.59 (0.66–3.86)
Sulfonylureas ^c^	2/658	664	2378/1,303,834	1,312,493			
Other ^c^	1/325	327	2379/1,304,166	1,312,830			
*Other infections*							
Metformin	108/1555	1617	74,272/1,269,264	1,311,540	1.19 (0.98–1.43)	1.13 (0.91–1.40)	1.09 (0.87–1.37)
Sulfonylureas	43/639	664	74,337/1,270,180	1,312,493	1.15 (0.85–1.55)	1.06 (0.76–1.46)	0.83 (0.53–1.31)
Other	26/312	327	74,354/1,270,507	1,312,830	1.42 (0.97–2.09)	1.33 (0.89–2.00)	1.26 (0.82–1.94)

(a) Adjusted for other types of non-insulin anti-hyperglycemic agents, mother’s age (≤30 years, 31–40 years, ≥41 years), father’s age (≤30 years, 31–40 years, ≥41 years), parity (1 or more than 1), sex of the child, maternal and paternal comorbidity, calendar year of birth (1997–2002, 2003–2008, 2009–2014, 2015–2018), and mothers’ use of antidiabetics in a regression model. (b) Adjusted for other types of non-insulin anti-hyperglycemic agents, mother’s age (≤30 years, 31–40 years, ≥41 years), father’s age (≤30 years, 31–40 years, ≥41 years), parity (1 or more than 1), sex of the child, mother’s BMI (<18.5, 18.5–24.9, 25–29.9, ≥30, kg/m^2^) maternal smoking in pregnancy (yes/no), maternal and paternal comorbidity and calendar year of birth (1997–2002, 2003–2008, 2009–2014, 2015–2018) and mother’s use of antidiabetics in a regression model. (c) Outcomes in those exposed are less than 5 and therefore no risk estimates calculated.

## Data Availability

Not applicable.
